# Comparison of SpO_2_ values from different fingers of the hands

**DOI:** 10.1186/s40064-015-1360-5

**Published:** 2015-09-29

**Authors:** Gokcen Basaranoglu, Mefkur Bakan, Tarik Umutoglu, Seniyye Ulgen Zengin, Kadir Idin, Ziya Salihoglu

**Affiliations:** Department of Anesthesiology and Reanimation, Bezmialem Vakif University Faculty of Medicine, Istanbul, Turkey

## Abstract

Pulse oximetry is a frequently used tool in anesthesia practice. Gives valuable information about arterial oxygen content, tissue perfusion and heart beat rate. In this study we aimed to provide the comparison of peripheral capillary hemoglobin oxygen saturation (SpO_2_) values among every finger of the two hands. Thirty-seven healthy volunteers from operative room stuffs between the ages of 18–30 years were enrolled in the study. They were monitored after 5 min of rest. After their non invasive blood pressure, heart rate, fasting time and body temperature were measured, SpO_2_ values were obtained from every finger and each of two hands fingers with the same pulse oximetry. All the SpO_2_ values were obtained after at least 1 min of measurement period. A total of 370 SpO_2_ measurements from 37 volunteers were obtained. The highest average SpO_2_ value was measured from right middle finger (98.2 % ± 1.2) and it was statistically significant when compared with right little finger and left middle finger. The second highest average SpO_2_ value was measured from right thumb and it was statistically significant only when compared with left middle finger (the finger with the lowest average SpO_2_ value) (p < 0.05). SpO_2_ measurement from the fingers of the both hands with the pulse oximetry, the right middle finger and right thumb have statistically significant higher value when compared with left middle finger in right-hand dominant volunteers. We assume that right middle finger and right thumb have the most accurate value that reflects the arterial oxygen saturation.

## Background

Peripheral capillary oxygen saturation (SpO_2_) measured by pulse oximeter, is a simple and reliable objective measurement in routine medical practice that approximates the level of oxygen in arterial blood. Measurements with this inexpensive and noninvasive method also provide heart rate and an indication of tissue perfusion (pulse amplitude). Low perfusion (due to hypothermia, low cardiac output, increased systemic vascular resistance, profound anemia or etc.), venous pulsations in a dependent limb, excessive ambient light or motion can cause pulse oximetry artifact. Also, carboxyhemoglobinemia, methemoglobinemia and intravenous dyes can cause false SpO_2_ readings (Butterworth et al. 2013; Chan et al. 2013; DeMeulenaere 2007). There is no information in the current literature about which finger could give the highest or the reliable recording of SpO_2_. In this prospective study, we tested in young healthy adults if there is a difference of between-fingers SpO_2_ values.

## Methods

After university ethics committee approval (No: 71306642/050-01-04) and written informed consent was obtained, healthy volunteers from operating room stuff, aging between 18 and 30 years were included in this study in July to August 2013. Volunteers, who were smokers, pregnant or menstruating, having ulnar or radial arterial failure due to Allen test results, having hypotension, bradycardia, anemia or hemoglobinopathy, have nail polish in the fingers, were excluded from the study.

Volunteers with an at least 8 h of fasting period were monitored after 5 min of resting. All SpO_2_ measurements were done in the same place and ambient light and the same brand monitor (GE B30 Medical system, Freiberg, Germany) was used in all volunteers. All SpO_2_ values were recorded in the sitting position and simultaneous blood pressure, heart rate and body temperature were noted. Measurements of each finger (abbreviations for fingers used in the text are shown in Table 1) were recorded after waiting at least 1 min.

**Table 1:** Abbreviations for fingers

Right thumb	R1
Right index finger	R2
Right middle finger	R3
Right ring finger	R4
Right little finger	R5
Left thumb	L1
Left index finger	L2
Left middle finger	L3
Left ring finger	L4
Left little finger	L5

### Statistical method

Repeated Anova test was used to compare measurements. If there was significant result a post hoc Bonferroni test was used to evaluate all multiple comparisons (p < 0.05 was considered as statistically significant).

## Results

A total of 370 SpO_2_ measurements obtained from 37 volunteers. Demographic data and hemodynamic results are shown in Table 2. Hypotension, hypothermia, tachycardia, bradycardia did not observed in none of the volunteers. There was no radial or ulnar artery insufficiency determined by Allen test, which was performed clinically. The average SpO_2_ values of each 10 finger were ranked as follows: R3 > R1 > R2 > R4 > L4 > L1 > L5 > R5 > L2 > L3 and listed in Table 3. Comparison of SpO_2_ values between fingers is shown in Table 3. Forty-five comparisons were done between fingers (Repeated Anova, F: 3.621, p = 0.004). The highest average SpO_2_ value was measured from R3 (98.2 % ± 1.2) and it was statistically significant when compared with R5 and L3. The second highest average SpO_2_ value was measured from R1 and it was statistically significant only when compared with L3 (the finger with the lowest average SpO_2_ value).

**Table 2:** Demographic data and hemodynamic values of volunteers

Age (years)	24.1 ± 2.7
Height (cm)	168 ± 9.4
Weight (kg)	64.7 ± 15.6
Gender (male/female)	13/24
Dominant hand (right/left)	35/2
Heart rate (beats/min)	79.6 ± 9.6
Systolic blood pressure (mmHg)	114 ± 12
Diastolic blood pressure (mmHg)	66 ± 9
Body temperature (°C)	36.3 ± 0.52
Fasting time (h)	11.78 ± 0.85

Numbers are mean ± standard deviation or number of volunteers

**Table 3:** Multiple comparisons of repeated anova by Bonferroni test

	SpO_2_ values (%)	R1	R2	R3	R4	R5	L1	L2	L3	L4
R1	98.08 ± 1.04	–								
R2	97.89 ± 1.41	NS	–							
R3	98,19 ± 1.2)	NS	NS	–						
R4	97.81 ± 1.50	NS	NS	NS	–					
R5	97.30 ± 1.39	NS	NS	0.028	NS	–				
L1	97.59 ± 1.17	NS	NS	NS	NS	NS	–			
L2	97.27 ± 1.64	NS	NS	NS	NS	NS	NS	–		
L3	97.05 ± 1.94	0.024	NS	0.016	NS	NS	NS	NS	–	
L4	97.68 ± 1.20	NS	NS	NS	NS	NS	NS	NS	NS	–
L5	97.51 ± 1.33	NS	NS	NS	NS	NS	NS	NS	NS	NS

Numbers are mean ± standard deviation or *P* values. *P* < 0.05: statistically significant. Repeated anova, F: 3.621, p = 0.004

*NS* not significant

## Discussion

According to our results in 35 volunteers with right hand dominance, R3 had the highest average SpO_2_ value with the pulse oximetry, while in two volunteers with left hand dominance; the L3 had the highest value.

In a survey of health care workers for monitoring pulse oximetry, index finger was selected by 80 % for SpO_2_ measurement (Mizukoshi et al. 2009). Index finger dominantly is fed from deep palmar arcus created from radial artery. But middle fingers receive both ulnar and radial artery blood supply. Mizukoshi et al. have investigated the most suitable finger for the measurement of the pulse oximetric monitoring. In this study, Perfusion Index (PI) value gave different results in each finger (ANOVA, p < 0.01) and the PI value of the middle finger was measured as the highest both during normoperfusion and hypoperfusion, but no remarkable difference was found in SpO_2_ values between fingers, which may be due to the insufficient number of subjects (20 volunteers). Also, right or left hand origin or hand-dominance was not documented in that study.

The difference of SpO_2_ recordings between different fingers may not be clinically important, but this knowledge may be valuable in conditions with poor peripheral perfusion. Dominant hand and higher perfusion may explain the highest value in R3. But, the explanation of the lowest value in L3 is a little complicated. In the non-dominant hand, the size of the finger may become a negative contributing factor that determines the SpO_2_ recording.

Higher perfusion in the middle finger seems reasonable to expect the highest and most accurate SpO_2_ value. According to the results of our study, we believe that the middle finger of the dominant hand has the highest and possibly the most accurate SpO_2_ measurements. The highest SpO_2_ value can be taken as the most accurate value that reflects the arterial oxygen saturation (SaO_2_). Because there may be contributing factors that can decrease the SpO_2_ recording measured by a pulse oximeter lower than SaO_2_, but there is no contributing factor that can increase the SpO_2_ recording higher than SaO_2_ (when a carbon monoxide poisoning like condition does not exist).

The main limitation of our study was the leak of arterial blood gas analysis during SpO_2_ measurements for determining the accurate value. Another limitation of our study was that, we did not have adequate left hand dominance volunteers. Further studies can be made with adequate number of left hand dominance volunteers or corroborated by arterial blood gas analyses and PI parameters.

In conclusion, SpO_2_ measurement from the fingers of the both hands with the pulse oximetry, the right middle finger and right thumb have statistically significant higher value when compared with left middle finger in right-hand dominant volunteers. We assume that right middle finger and right thumb have the most accurate value that reflects the arterial oxygen saturation.
